# Regulation of Biobanks in Italy

**DOI:** 10.3389/fped.2020.00415

**Published:** 2020-09-02

**Authors:** Nunzia Cannovo, Mariano Cingolani, Rosa Guarino, Piergiorgio Fedeli

**Affiliations:** ^1^Federico II University Ethics Committee, Naples, Italy; ^2^Department of Law, Institute of Legal Medicine, University of Macerata, Macerata, Italy; ^3^University of Federico II, Naples, Italy; ^4^Legal Medicine Section, School of Law, University of Camerino, Camerino, Italy

**Keywords:** biobanks, Italy, genetics research, regulation, COVID-19

## Abstract

In Italy, a biobank is “a non-profit organization that must be officially recognized by the appropriate healthcare authority in the member states and must guarantee the treatment, distribution and conservation of biological material according to standards of quality and professionalism,” but must not conserve material already regulated by specific laws, as is the case for organs for transplants, blood for transfusions, as well as embryos and gametes for medically assisted reproduction. The concept of biobank includes not only biological samples, but also the related database of clinical and personal information, from which the subject's lifestyle can be deduced. Unfortunately, at the moment, Italian law does not offer specific itineraries for achieving this legal status.

## Introduction

The phenomenon of biobanks is relatively recent (1), and there has been a very sharp increase in the establishment of national and international collections of biosamples in public hospitals and private structures.

As noted by Fedeli et al. (2), different definitions of “biobank” have been proposed in the national and international literature for this multifaceted phenomenon. It can be said that a biobank is “a structured collection of human biological material accessible on the basis of certain criteria” (3, 4), “in accordance with a code of good practice and correct behavior and with further indications provided by ethics committees and universities” (5) and “in which the information contained in the biological material can be traced to a specific person” (2), “for diagnostic, treatment and research purposes” (6).

Beginning in 1994, the European Union has provided regulations for biobanks (7), defining the parameters of quality and safety for donation, procurement, analysis, processing, storage and distribution of human tissues and cells, stipulating the adoption of necessary measures of protection of data, including genetic data, and other measures for safeguarding information collected in the context of activities of donation, procurement, monitoring, processing, conservation, storage and distribution of human tissues and cells to be used for applications in humans, as well as products derived from human tissues and cells, to be used in applications in humans (art. 14) (8). In addition, the EU has provided technical prescriptions (9) for the donation, procurement and monitoring of human tissues and cells, and has clarified the distinction between a disease biobank and research biobanks (10).

In Italy, given the delay in the passage of a national law, the Regions have taken the initiative to complete local legislative itineraries to re-organize the sector.

The authors provide an overview of Italian legislation on research biobanks.

## Biobanking: Ideological Aspects

The concept of biobank includes not only biological samples, but also the related database of clinical and personal information, from which the subject's lifestyle can be deduced. Thus, the particular nature of a biobank is due to the fact that it contains genetic information that can be traced to the subjects who provided the biological material (11).

The problem of the juridical conception of biological samples for medical research lies in their two-sided nature as collections of cells and as sources of health and genetic data (12). With emphasis on the exquisitely material nature of these samples, intense debate has revolved around the ownership of biological samples (13–16), and the legal relationship that binds the subjects to the samples, when the latter have been separated from their bodies.

For some authors (17), when diagnostic or treatment procedures call for the ablation of material or parts from the body, even though the procedure does not cause a permanent reduction of physical integrity, these materials or parts are in effect separated from the body, and as such acquire the nature of disposable personal property (art. 810 c.c.), within the limits of legal provisions already in vigor (art.5 c.c.) and are the object of ownership just as any other good (18–23). The most recent thought, however, has confuted the existence of a ius in se ipsum, arguing that after the separation, the body parts become external things, and thus can be owned. Thus, there is a foundation for the claim that these tissues have been abandoned and that they can be taken by those who are interested in using them, because “for the most part, they offer the persons neither an interest of use nor an interest of exchange,” and consequently, with the separation, these goods become res nullius (24) as derelict or abandoned, and thus can be acquired (25, 26). A certainly original theory draws a parallel between the law on separated parts of the body and that on works of creativity, based on the concept that just as a subject owns works of his/her creativity, so the individual should be deemed the owner of his/her own biological material (art. 2576 cc). According to this juridical construction, the part removed is a res that was created by the subject, albeit with the help of the surgeon, and therefore the subject should be the sole owner (25).

The Oviedo Convention (art. 21) (27) re-affirmed "the prohibition of financial gain” (28–35), for biological samples. There are two possible interpretations of this principle, a more radical one and a more permissive one. An initial reading (36) excludes a priori any possibility of constituting patrimonial rights over the body and human tissues even after their separation from the body. The more permissive theory (32) refers to the impossibility of using individual parts of the body for gain, ensuring the freedom and spontaneity of donations on the basis of the rule of the extra-patrimonial nature of the circulation of rights over the human body. However, the solution is not easy, because the relationship of ownership entails a series of rights and faculties that must subsist in order to claim full ownership of something; clearly, this cannot be the case with biological samples, which are considered goods extra commercium, and cannot be disposed of for gain, because this would be an affront to human dignity, the protection of which is grounded in the Constitution of the Italian Republic (art. 2).

This theme inevitably intertwines with the profile of the allocation of ownership of biological samples when the patient has given consent for their conservation and use (37).

Allocation of ownership to donors would legitimize a sort of commodification of the body, which could undermine scientific research and limit the range of experimentation, given the possibility that subjects may request the destruction of the samples at any time (38). Instead, researchers are able to gain useful information from the study of these materials, as they have the technical skills needed to exploit the biological characteristics of the samples and above all to obtain information from them. However, the attribution of exclusive ownership to researchers would inevitably exclude the “donors” from participating in the biotechnological research and its profits, with the risk of creating an irreparable breach in the alliance between medical science and the community, which was established when the biological material was freely granted. Also, if ownership was granted to research institutions, there could ensue a fratricidal race to “grab up” biological materials and use them for profit.

Thus, a new understanding of the biological sample is emerging. What was once considered special waste produced during surgery is now viewed as an irreplaceable source of medical and genetic information, an inexhaustible source for the development of biomedical science. Samples are instruments of biological identity (39, 40) inasmuch as they identify the body from which they came through the genetic patrimony of that person, “pieces of each subject, conserved in the very numerous databanks where the identity of the subject is sectioned and taken apart” (41), since samples can symbolically be considered a “crystal ball” (42), a vehicle that provides genetic data, through which future health conditions can be predicted (43). In fact, from the point of view of information, “the separation of the biological sample does not mean that it has complete autonomy from the body-subject, but only that it may have autonomous circulation” (42).

Thus, the very particular nature of these samples appears evident: they have a material dimension, shaped by ownership law, and a dimension of information, expression of the personhood and identity of the subject (41, 44, 45). The perplexities arise from the entanglement of the uncontestable benefits that access to such data can bring to scientific evolution, and the incontrovertible need to protect the individual (46). On the one hand, subjects risk damage to their right to privacy (47); if others can access their individual results, this may lead to new forms discrimination (48) and social injustice (49). In such situations, genetic information can be viewed as having “multiple meanings” and being “dangerous” and “ambivalent” (50). On the other hand, these results are of enormous importance for scientific research, as articulated in the Declaration on the human genome (1997) (51) according to which, “benefits from advances in biology, genetics and medicine, concerning the human genome, shall be made available to all, with due regard for the dignity and human rights of each individual. Freedom of research, which is necessary for the progress of knowledge, is part of freedom of thought. The applications of research, including applications in biology, genetics and medicine, concerning the human genome, shall seek to offer relief from suffering and improve the health of individuals and humankind as a whole” (art. 12).

## National Legislative Framework

In Italy, biobanks are defined as “service units, without direct profit-making purposes, for the collection and conservation of human biological material used for diagnostic purposes, for studies of biodiversity and for research” (52). Thus, a biobank is “a non-profit organization that must be officially recognized by the appropriate healthcare authority in the member states and must guarantee the treatment, distribution and conservation of biological material according to standards of quality and professionalism” (6), but must not conserve material already regulated by specific laws, as is the case for organs for transplants, (53) blood for transfusions (54), as well as embryos and gametes for medically assisted reproduction (55).

In Italy, most of the biobanks and centers of biological resources are found at structures or institutions that are part of or connected to the national healthcare service. Consequently, the biological samples (sputum, serum, plasma, tissue, etc.) should be collected and stored according to international standard operating procedures and those developed *ad hoc* (56–58).

## Legal and Administrative Requirements for Collection and Use

A biobank that intends to leave behind its nature as a volunteer, not-for-profit entity, must be accredited or at least certified. Unfortunately, at the moment, Italian law (59) does not offer specific itineraries for achieving this legal status, and it is necessary to refer to the forms of self-regulation adopted by biomedical laboratories, non-binding acts, and European and international law, where regulations for the phenomenon have already been established. Though shared standards are lacking (60), it is important that the following criteria must be respected for the establishment of a biobank: systematic organization; accessibility (indicate whether or not it is open to third parties); primary purpose and directions of the research/use to be undertaken with the samples; what information will be kept; types of samples/data (living organisms and/or DNA and/or sources of DNA or information based on DNA), with a clear separation between biological materials to be used for treatments and those to be used for research (61). Regarding accreditation, in line with the indications of the Conference of the Regions and the National Government, the individual Regions will take responsibility, following the unified lines of conduct indicated by the national government, especially the standards of safety and quality control.

European Directive 2004/23 of March 31 2004 (62) and the subsequent 2006/17 (63) introduced and clarified the need to ensure traceability of donated tissues and cells, through the assignment of a unique code to each donation and each of the products associated with it. Regarding traceability, the Ministerial Decree for Productive Activities of June 26, 2006 introduced the procedures for the certification of Biobanks as Centers of Biological Resources, while D.Lgs 191/07, which introduced tissue institutions for the conservation of cells and tissues for treatment purposes, transposed the provisions on quality and safety for donation, procurement, monitoring, processing, conservation, storage and distribution of human tissues and cells, which could have an interesting implementation also in the management of biobanks, in an initial phase of lack of *ad hoc* legislation.

This legislative decree stipulates that tissue institutes must conserve the data necessary to ensure traceability in all phases, for at least 30 years after clinical use, also in digital format.

A recent Italian law (Law 3/2018) (64) allowed the collection of biological samples for research purposes. Specifically, it made possible the use of biological material from previous diagnostic or treatment activities, or kept for any other purposes, on the condition of obtaining the patient's informed consent beforehand. A subsequent law, (Legislative decree n. 52/2019) (65) assigned the task of defining the criteria for collecting biological samples to the Higher Institute of Healthcare.

As noted by Cannovo et al. (66), the Guarantor for the Protection of Personal Data allows genetic data and biological samples (67) collected for scientific and statistical research to be communicated or transferred to research entities and institutes, associations and other research-oriented public and private organizations, exclusively in the context of joint projects. However, these data and samples, with all personal data removed, can also be made available to third parties not participating in joint projects, for scientific purposes directly connected to those for which they were originally collected, and clearly determined in the written request for the data/samples. In this case, the subject making the request must commit to not using the data and/or samples for purposes different from those indicated in the request, and to not communicating them or transferring them to other parties.

A provision established by the Guarantor for the Protection of Personal Data regulates the acquisition of informed consent. When every reasonable effort has been made to contact the subject for consent, but for particular reasons they have not met with success, the Guarantor gives an active role to Ethics Committees (67).

## Conclusion

The importance that the complex phenomenon of biobanks has gained in recent years is intrinsically connected to the incessant advances in knowledge about genetics and the importance of the human genome, a special and irreplaceable source of genetic and medical data useful for the development of medical science (68).

The treatment of genetic data requires and justifies a particular legal safeguarding, but there is a need for juridical regulations that, far from limiting the freedom of research, direct the concrete developments to foster and protect the well-being and absolute value of the human person (59) with thoughtful balancing of principles, in order to ensure respect for the rights of subjects who choose to donate their samples, and also those of the researchers and institutions that intend to use them for their scientific projects.

Given the fragmented and inconsistent juridical situation in Italy, we believe it would be wise to consider biological samples as commons (42), that is, community property, or in other words, supra-individual goods at the service of the community. This should become a cardinal value in order to ensure the functionalization of these biological materials for scientific research purposes and their distribution among researchers in a democratic and transparent way. This would make it possible to disentangle the difficult knot of the dual nature of biological material, and to exploit in the best way possible the rights of intellectual property that can be obtained from it, using the model of biobanks as Biotrusts, that is, third subjects, in addition to the patients and researchers. In this sense, the biobank would serve as a repository for the conservation and safekeeping of biological materials and as a guarantor of the principles underlying them.

## Data Availability Statement

The original contributions presented in the study are included in the article/supplementary material, further inquiries can be directed to the corresponding author/s.

## Author Contributions

Material preparation, data collection, and analysis were performed by NC, RG, and PF. The first draft of the manuscript was written by NC. MC has contributed in revising the manuscript. All authors commented on previous versions of the manuscript, read and approved the final manuscript, and contributed to the study conception and design.

## Conflict of Interest

The authors declare that the research was conducted in the absence of any commercial or financial relationships that could be construed as a potential conflict of interest.
